# Cooperation of Hsp70 and Hsp100 chaperone machines in protein disaggregation

**DOI:** 10.3389/fmolb.2015.00022

**Published:** 2015-05-19

**Authors:** Axel Mogk, Eva Kummer, Bernd Bukau

**Affiliations:** Center for Molecular Biology of the University of Heidelberg (ZMBH) and German Cancer Research Center (DKFZ), DKFZ-ZMBH AllianceHeidelberg, Germany

**Keywords:** Hsp70, Hsp100, protein disaggregation, chaperone, AAA+ proteins

## Abstract

Unicellular and sessile organisms are particularly exposed to environmental stress such as heat shock causing accumulation and aggregation of misfolded protein species. To counteract protein aggregation, bacteria, fungi, and plants encode a bi-chaperone system composed of ATP-dependent Hsp70 and hexameric Hsp100 (ClpB/Hsp104) chaperones, which rescue aggregated proteins and provide thermotolerance to cells. The partners act in a hierarchic manner with Hsp70 chaperones coating first the surface of protein aggregates and next recruiting Hsp100 through direct physical interaction. Hsp100 proteins bind to the ATPase domain of Hsp70 via their unique M-domain. This extra domain functions as a molecular toggle allosterically controlling ATPase and threading activities of Hsp100. Interactions between neighboring M-domains and the ATPase ring keep Hsp100 in a repressed state exhibiting low ATP turnover. Breakage of intermolecular M-domain interactions and dissociation of M-domains from the ATPase ring relieves repression and allows for Hsp70 interaction. Hsp70 binding in turn stabilizes Hsp100 in the activated state and primes Hsp100 ATPase domains for high activity upon substrate interaction. Hsp70 thereby couples Hsp100 substrate binding and motor activation. Hsp100 activation presumably relies on increased subunit cooperation leading to high ATP turnover and threading power. This Hsp70-mediated activity control of Hsp100 is crucial for cell viability as permanently activated Hsp100 variants are toxic. Hsp100 activation requires simultaneous binding of multiple Hsp70 partners, restricting high Hsp100 activity to the surface of protein aggregates and ensuring Hsp100 substrate specificity.

## Introduction

Maintenance of protein homeostasis (proteostasis) under a large variety of environmental stress conditions, such as exposure to heat, or upon intrinsic perturbation of the proteome, is a central achievement of cells critical to physiology and survival of organisms (Morimoto, 2011). Proteostasis is achieved by an efficient and adaptive protein quality control system that detects non-functional and potentially harmful misfolded proteins, which are prone to aggregation, and promotes their refolding by chaperones and degradation by ATP-dependent proteases. Severe or persistent stress exceeding the capacity of this system results in increased protein aggregation, which is associated with pathophysiological states of cells and cell death. The exposure to changing environmental growth conditions is particularly severe in case of unicellular and sessile organisms. To reverse protein aggregation, bacteria, fungi and plants encode a powerful bi-chaperone system composed of two cooperating ATP-driven machines: the hexameric AAA+ chaperone Hsp100 (ClpB in *Escherichia coli*, Hsp104 in *Saccharomyces cerevisiae*) acting as protein disaggregase, and the Hsp70 chaperone system (DnaK-DnaJ-GrpE (KJE) in *E. coli*, Ssa1-Ydj1/Sis1-Sse1/Fes1 in *S. cerevisiae*). The bi-chaperone system solubilizes and reactivates a broad range of aggregated proteins (Glover and Lindquist, 1998; Goloubinoff et al., 1999; Motohashi et al., 1999; Zolkiewski, 1999), conferring thermotolerance and ensuring cell survival under severe stress conditions (Sanchez and Lindquist, 1990; Squires et al., 1991; Hong and Vierling, 2000; Queitsch et al., 2000). In this review we will focus on the working principle of Hsp100 proteins and how their activities are controlled and modulated by the Hsp70 partner.

## Hsp100 proteins: threading machines for protein unfolding

**Figure 1:**
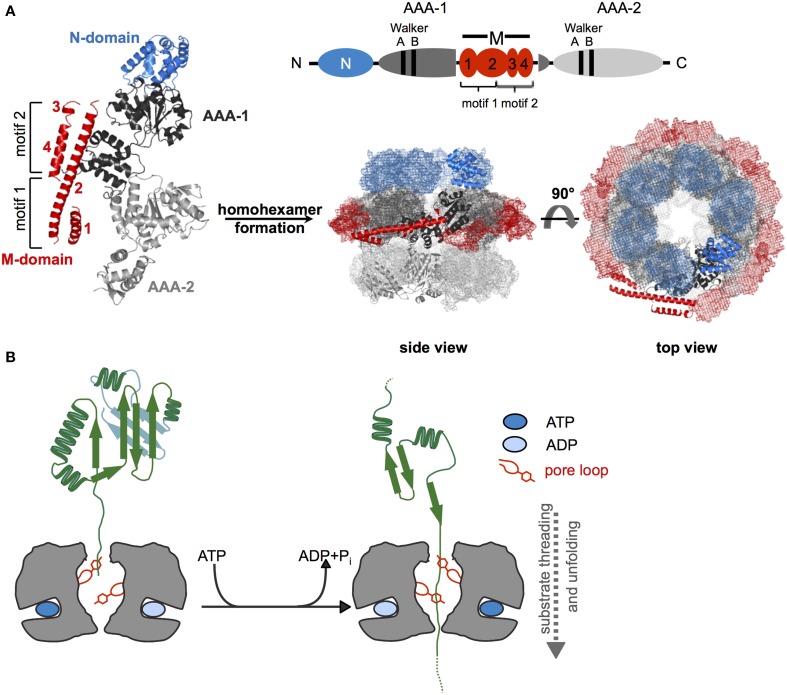
**Structure and basic threading mechanism of ClpB/Hsp104 disaggregases. (A)** Domain organization, structure, and hexameric model of ClpB/Hsp104. The ClpB/Hsp104 protomer consists of an N-terminal (N) domain, two AAA domains (AAA-1, AAA-2) encompassing conserved Walker A and B motifs for ATP binding and hydrolysis, and an inserted ClpB/Hsp104-specific coiled-coil middle (M) domain. The M domain consists of four α-helices that are numbered accordingly. Motif1 comprises helices 1 and 2; motif2 consists of helices 2, 3, and 4. The monomer assembles into a hexamer consisting of three layers (rings) formed by N-domains, AAA-1/M-domains, and AAA-2 domains. A model of *E. coli* ClpB hexamers based on negative staining EM is given (Carroni et al., 2014). **(B)** Depiction of Hsp100 threading activity. Conserved aromatic residues (Tyr) are located on mobile loops at the central pore of the hexamer and contact substrate proteins. ATP binding and hydrolysis lead to conformational changes of loop segments, generating a pulling force that is linked to substrate unfolding and threading.

Hsp100 chaperones are ATP fueled unfolding machineries belonging to the AAA+ protein superfamily (Neuwald et al., 1999). AAA+ proteins share the AAA domain, which is defined by a region of ~230 amino acids in length, comprising conserved Walker A and Walker B motifs for nucleotide binding and hydrolysis. The AAA domain is formed by a RecA-like large subdomain and an α-helical small subdomain, providing the ATP binding site at their subdomain interface. The AAA domain also drives protein oligomerization, usually into hexameric ring-like structures with a central pore (Figure 1A).

Hsp100 chaperones differ in the number of AAA domains (one or two) per protomer and the presence of extra domains, which provide functional specificity by controlling substrate interactions. ClpB/Hsp104 consist of two AAA domains (referred to as AAA-1 and AAA-2), which are oriented head-to-tail and two additional domains: an N-terminal domain (N-domain) and a middle domain forming a coiled-coil structure (M-domain) that is inserted in the first AAA domain (Figure 1A). Functions and positions of these extra domains will be discussed in later chapters.

Hsp100 proteins exert an ATP-driven threading activity and translocate protein substrates through their central channel (Figure 1B). This threading activity is used by Hsp100 proteins that form proteolytic complexes with peptidases (e.g., *E. coli* ClpA or ClpX with the ClpP peptidase) to feed substrates into the associated proteolytic chamber for degradation. ClpB and Hsp104 do not associate with peptidases and use their threading power to disentangle single polypeptide chains trapped within protein aggregates.

Threading can be initiated at substrate N- or C-termini or at internal sites and even concurrent translocation of two polypeptides (e.g., a looped polypeptide) through the pore is possible (Burton et al., 2001; Haslberger et al., 2008). Mobile pore loops harboring conserved aromatic residues are crucial for substrate translocation by Hsp100 proteins. These loop structures can move downwards along the central translocation channel in a nucleotide-dependent manner (Figure 1B). Through direct contacts with substrates such movements transmit a pulling force leading to substrate transport across the channel. Mutations in ClpB and Hsp104 pore loops strongly reduce or abrogate protein disaggregation and aggregated proteins can be crosslinked to the central translocation channel during ongoing protein disaggregation (Lum et al., 2004; Schlieker et al., 2004; Weibezahn et al., 2004).

Substrate translocation by bacterial AAA+ chaperones (ClpX) happens in discrete steps of 5–8 amino acids, consuming one ATP molecule per step (Aubin-Tam et al., 2011; Maillard et al., 2011). Axial staggering of the pore loops facilitates substrate handover between the loops and prevents substrate backsliding (Glynn et al., 2009). A denaturation force of up to 20 pN is iteratively applied on folded substrates when initial threading of a polypeptide segment pulls the remaining part of the substrate against the narrow entrance pore of the Hsp100 hexamer. The pulling will disrupt local structural elements and the success of substrate unfolding depends on local but not global substrate stability (Lee et al., 2001).

## Hsp100 proteins form asymmetric AAA rings

How ATP hydrolysis is orchestrated and linked to the formation of a mechanical force is key to understand Hsp100 function. Early studies revealed stimulation of ATPase activity by substrate (Woo et al., 1992) and demonstrated allosteric communication between ClpB/Hsp104 ATPase domains (Schlee et al., 2001; Hattendorf and Lindquist, 2002). The ATPase domains must interact with each other since mutations in one AAA domain affects ATP hydrolysis and chaperone activity of the other (Schirmer et al., 2001; Watanabe et al., 2002; Mogk et al., 2003). ClpB/Hsp104 ATPase regulation is highly complex as it involves intra- and intermolecular communications within each and between the two ATPase rings, and their control via the ClpB/Hsp104-specific M-domain as discussed later. We will therefore first describe findings for the Hsp100 family member ClpX, which harbors only one ATPase domain and allowed to unravel basic principles of Hsp100 ATPase regulation.

**Figure 2:**
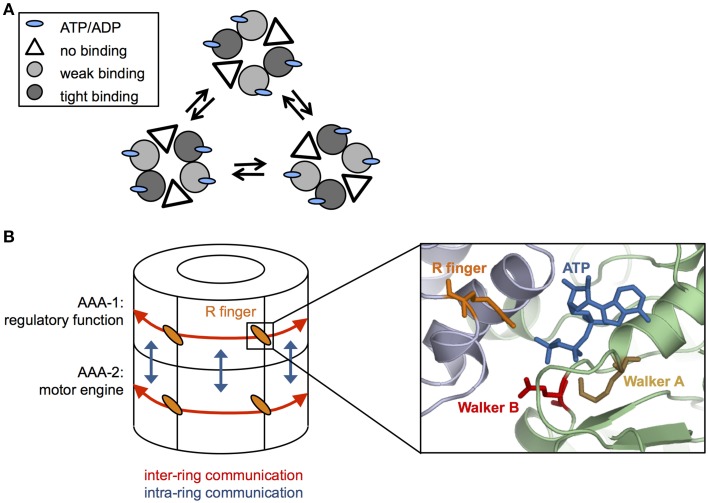
**Coordination of ATP hydrolysis in Hsp100 proteins. (A)** Cycling of ATP loaded and empty units in a hexameric ClpX ring. ClpX hexamers are composed of subunit pairs that exhibit weak or tight binding to nucleotide or remain unbound. ATP hydrolysis triggers conformational changes leading to interchange of the different subunit pairs **(B)** Simplified cartoon of communications within (red arrows) and between (blue arrows) AAA-1 and AAA-2 rings in ClpB/Hsp104. Cooperativity within each AAA ring is mediated by conserved arginine fingers that are located at the subunit interfaces, contacting the γ-phosphate of ATP bound at an adjacent subunit and allowing to signal nucleotide states across the ring. Positions of conserved glutamate and lysine residues of Walker A and B motifs, respectively, ATP and arginine fingers are given based on (Lee et al., 2007). AAA-1 has mainly a regulatory function and controls ATP turnover at AAA-2, which represents the main motor engine for substrate threading.

The ClpX oligomer forms a proteasome-like complex with the peptidase ClpP (for comprehensive review see Baker and Sauer, 2012). Homohexameric ClpX is an asymmetric assembly as only four out of six nucleotide binding sites are occupied (Hersch et al., 2005; Glynn et al., 2009) (Figure 2A). The AAA+ proteins PAN and HslU also only bind four ATP molecules under saturating nucleotide concentrations, indicating that the existence of loaded and empty nucleotide binding sites is a common feature of this protein family (Yakamavich et al., 2008; Smith et al., 2011).

Structural differences among nucleotide-bound ClpX subunits indicate the existence of weak and tight binding states (Glynn et al., 2009; Stinson et al., 2013). The different nucleotide states (empty, weak, and tight nucleotide binding) dynamically interconvert in a pairwise and synchronous fashion (Stinson et al., 2013) (Figure 2A). Cycling of different conformational states demands for nucleotide sensing and allosteric communication between AAA domains. The ATPase active sites are located at the interface of two neighboring subunits, offering a pathway for signal transmission. Changes in nucleotide occupancy could be transmitted through the subdomain connecting hinge region, which also contacts the bound nucleotide, resulting in rigid body movements of large and small AAA domains of neighboring subunits that are tightly packed (Glynn et al., 2009). Surprisingly, ClpX hexamers with only a single active ATPase subunit still exhibit ATPase and threading activities (although weak) indicating that individual subunits can work independently and ATP hydrolysis can proceed in a probabilistic manner (Martin et al., 2005). However, in ClpX wild type hexamers ATP hydrolysis and resulting conformational changes are coordinated, involving two to four ATPase subunits (Sen et al., 2013). This coordinated threading activity leads to power strokes with higher strength that are linked to more efficient substrate translocation in distinct, 2–4 nm steps (Sen et al., 2013).

It needs to be determined whether the regulatory principles determined for ClpX also hold true for other AAA+ proteins and ClpB/Hsp104. In agreement with findings for ClpX only 8 out of 12 nucleotide binding sites are occupied in ClpB and mutant analysis suggests that partial nucleotide occupancy is an intrinsic and independent property of each ClpB AAA domain (Fernandez-Higuero et al., 2011; Carroni et al., 2014). Mixing experiments of crosslinked dimeric ClpB subunits (wt or ATPase deficient) suggest that binding of three ATP molecules in one AAA ring is not sufficient for stimulation of nucleotide hydrolysis, suggesting a tighter coupling of ATPase domains as determined for ClpX (Yamasaki et al., 2015). Four ClpB subunits are suggested to build a cooperative unit, which is reflected in cooperative nucleotide binding, substrate interaction at AAA-1, and protein disaggregation (determined Hill-coefficients *n* ≈ 4) (del Castillo et al., 2010; Fernandez-Higuero et al., 2011).

## Arginine fingers mediate allosteric communications within AAA rings

How is cooperativity in nucleotide binding and hydrolysis achieved within the ClpB/Hsp104 ring? Highly conserved arginine residues, termed arginine fingers, contact the γ-phosphate of ATP bound in neighboring subunit (Figure 2B), thereby providing a structural framework to sense nucleotide states and to transmit this information across the ring. In ClpB/Hsp104 arginine fingers of each AAA domain mediate cooperativity of ATP binding and hydrolysis in an allosteric fashion (Zeymer et al., 2014) and are essential for disaggregation activity and ATP hydrolysis in the respective AAA ring (Mogk et al., 2003; Yamasaki et al., 2011; Biter et al., 2012).

While cooperativity exists within each AAA ring of ClpB/Hsp104 it is crucial to understand how the individual rings communicate. Hsp104 has been suggested to function as a two-stroke motor showing inverse activities and reciprocal regulation of the AAA-1 and AAA-2 domains (Franzmann et al., 2011). For instance ATP-binding at AAA-1 inhibits ATP hydrolysis by other AAA-1 domains but stimulates ATP turnover at AAA-2 (Franzmann et al., 2011). AAA-1 domains are therefore predicted to largely fulfill a regulatory function, coupling ATP-dependent substrate binding to high ATP turnover at AAA-2 (Figure 2B). Consistent with this idea the prion curing agent guanidinium hydrochloride (GdmHCl) inhibits ClpB and Hsp104 activity by binding close to the ATPase center of AAA-1 (Zeymer et al., 2013) and inhibiting continuous ATP turnover at this site (Grimminger et al., 2004; Kummer et al., 2013; Zeymer et al., 2013). AAA-2 is suggested to represent the main motor engine for substrate threading and in agreement with this model ClpB and Hsp104 pore loop or ATPase mutants at AAA-2 exhibit stronger disaggregation defects compared to their AAA-1 counterparts (Hattendorf and Lindquist, 2002; Mogk et al., 2003; Lum et al., 2004; Weibezahn et al., 2004).

The dynamics of ClpB/Hsp104 oligomers adds further complexity to ATPase control (Werbeck et al., 2008). ATP hydrolysis is decreasing ClpB/Hsp104 hexamer stability, suggesting that subunit exchange is happening during ongoing disaggregation (Aguado et al., 2015). This is supported by the immediate poisoning of ClpB-wt disaggregation activity upon addition of mutant subunits, which rapidly form mixed oligomers with wild type subunits (Haslberger et al., 2008; Werbeck et al., 2008). Oligomer dynamics might prevent jamming of the ClpB/Hsp104 oligomer by stable protein domains of aggregated substrates that cannot be further processed (Haslberger et al., 2008; Werbeck et al., 2008; Aguado et al., 2015). Indeed, ClpB solubilizes aggregated proteins harboring tightly folded domains without unfolding them, indicating that partially threaded protein substrates are released upon oligomer dissociation (Haslberger et al., 2008).

## Hsp100 recruitment via Hsp70 binding to the Hsp100 M-domain

**Figure 3:**
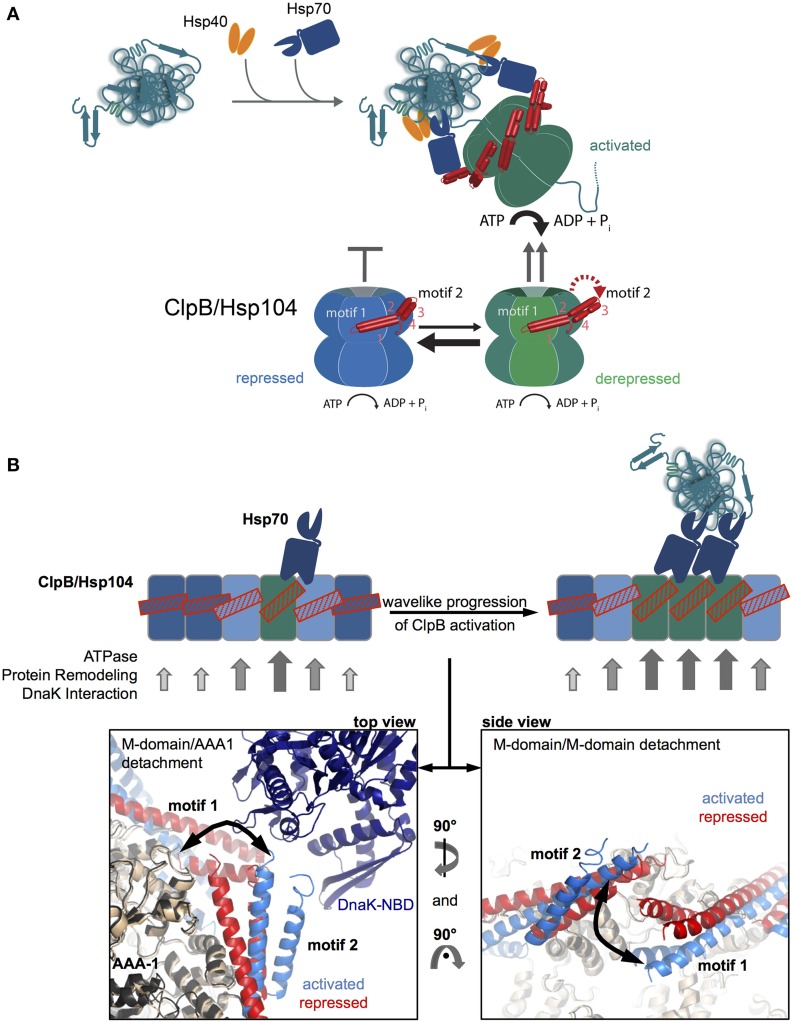
**M-domains control ClpB/Hsp104 activity and Hsp70 cooperation. (A)** Disaggregating chaperones bind in a hierarchical order to protein aggregates involving Hsp40 binding as first and ClpB/Hsp104 recruitment as latest step. Hsp70 activates ClpB/Hsp104 at the surface of protein aggregates. ClpB/Hsp104 activity is controlled by its unique M domain. M-domain attachment to AAA-1 represses ClpB/Hsp104 motor activity (repressed state). Detachment of motif2 from AAA-1 (derepressed state) and subsequent substrate engagement lead to full ClpB activation (activated state). Hsp70 only interacts with detached M-domains, shifting the equilibrium from repressed to derepressed activity states of ClpB/Hsp104 and coupling ClpB/Hsp104 recruitment to activation. **(B)** Wavelike progression of ClpB/Hsp104 activation upon an initial Hsp70 encounter. Cartoon of an opened-out ClpB/Hsp104 ring with different M-domain conformations. Horizontal M-domain positions are stabilized by intermolecular contacts between motif1 and motif2 of adjacent M-domains and keep ClpB/Hsp104 in a repressed state (bluish subunits). Tilted M-domain positions break intermolecular M-domain contacts liberating M-domain motif2 for Hsp70 interaction and causing ClpB/Hsp104 activation (greenish subunits). Stabilization of tilted M-domain conformations by Hsp70 binding favors activation of adjacent subunits, and the active cluster can move around the ring in a wave-like manner. Hexameric models of repressed and activated ClpB variants highlighting M-domain positions and interactions are shown (Carroni et al., 2014). DnaK docking to M-domain motif2 is based on (Rosenzweig et al., 2013).

The basal characterization of the disaggregation reaction revealed that Hsp70 acts prior to Hsp100 in the disaggregation process (Weibezahn et al., 2004; Zietkiewicz et al., 2004). Further, analysis showed that Hsp70 already controls the very initial step of Hsp100 activity, namely the binding to protein aggregates (Acebron et al., 2009; Winkler et al., 2012) (Figure 3A). The ClpB and Hsp104 homologs have almost no ability to recognize aggregated proteins *in vivo* in absence of Hsp70. How does Hsp70 recruit Hsp100 to its substrates?

The observation of species-specific cooperation of ClpB with DnaK and Hsp104 with Ssa1 in protein disaggregation suggested direct physical contacts between the partners and argued against independent and sequential activities (Glover and Lindquist, 1998; Krzewska et al., 2001). The ClpB/Hsp104-specific M-domains were shown to mediate species specificity (Sielaff and Tsai, 2010; Miot et al., 2011), explaining their essential roles in protein disaggregation (Kedzierska et al., 2003; Mogk et al., 2003) and suggesting a role in Hsp70 binding. The M-domain coiled-coil structure is composed of four helices forming two wings termed motif1 and motif2 (Lee et al., 2003) (Figure 1A). Crosslinking approaches revealed direct binding of DnaK to M-domain motif2 of ClpB (Seyffer et al., 2012; Lee et al., 2013). The DnaK-ClpB interaction was subsequently revealed at atomic resolution by NMR spectroscopy, showing that the M-domain motif2 binds to the ATPase domain of DnaK (Rosenzweig et al., 2013) (Figure 3B). The interaction surface overlaps with the binding interface of DnaK and its nucleotide exchange factor GrpE (Rosenzweig et al., 2013), rationalizing previous findings that disaggregation does not require GrpE assistance. Nucleotide exchange factors allow for release of Hsp70-bound substrates upon displacement of ADP and ATP rebinding. How is then an Hsp70-bound aggregated substrate released if GrpE cannot bind? ClpB binding does not accelerate nucleotide release from Hsp70 but substrate threading by the Hsp100 dissociates the substrate from the Hsp70 substrate binding domain thereby indirectly replacing GrpE function (Rosenzweig et al., 2013).

## Hsp100 M-domains link Hsp70 interaction to AAA ring activation

Do M-domains function as a mere binding platform of Hsp70? Early findings that M-domains interact with AAA-1 in a nucleotide-dependent manner and that M-domain mutations cause deregulated high ATPase activities suggested an additional function in controlling the ClpB/Hsp104 ATPase cycle (Haslberger et al., 2007). A thorough analysis of ClpB M-domain variants led to the identification of two distinct mutant classes (Oguchi et al., 2012; Seyffer et al., 2012). Repressed M-domain mutants rendered ClpB inactive in protein disaggregation and exhibited a tighter interaction between M-domain motif2 and AAA-1. Activated M-domain variants were superior in protein disaggregation, showed loss of M-domain motif2/AAA-1 interaction and exhibited high ATPase activity in presence of substrate. Comparable findings were obtained for related Hsp104 M-domain variants (Lipinska et al., 2013). These findings indicate that M-domains function as a molecular toggle to negatively regulate ClpB/Hsp104 ATPase and disaggregation activities (Figure 3A).

Further, understanding of the molecular basis of M-domain function was hampered by conflicting ClpB/Hsp104 oligomer reconstructions based on cryo electron microscopy. The oligomeric models differed particularly with respect to M-domain positioning, as the M-domain was either suggested to project outwards from the AAA ring (Lee et al., 2003, 2007) or to intercalate between AAA subunits (Wendler et al., 2007, 2009). Both models also could not explain M-domain function in controlling ClpB/Hsp104 activity. A recent cryo EM reconstruction determined a different position of M-domains, which nestle at the surface of the AAA-1 ring (Carroni et al., 2014). M-domains are contacting neighboring AAA-1 domains via motif1, while motif2 makes intrasubunit AAA-1 interactions. Furthermore, adjacent M-domains interact in a head-to-tail manner via motif1-motif2 contacts (Carroni et al., 2014) (Figure 3B). This M-domain position is in accordance with a multiplicity of biochemical analysis of ClpB/Hsp104 variants and is further substantiated by site-specific crosslinking and FRET analysis (Oguchi et al., 2012; Carroni et al., 2014). The intermolecular M-domain interactions also explain the previously noticed stabilizing effect of M-domain on ClpB assemblies (Kedzierska et al., 2003; del Castillo et al., 2011).

An asymmetric cryo EM reconstruction of ClpB hexamers revealed structural plasticity of M-domains, which exist in variable conformations, horizontal and tilted, that can be attributed to repressed and activated states. In the repressed state M-domain motif2 tightly interacts with the AAA-1 ring whereas it is entirely solvent exposed and flexible in the activated state. The various M-domain conformations exist only transiently, constantly drifting between repressed and activated ones. The activated state must therefore be short-lived and most ClpB subunits exist in repressed states at a given time (Figure 3A). Importantly, only the dissociated state of M-domains motif2 allows for Hsp70 binding, which is not possible if M-domain motif2 is engaged in interaction with motif1 of an adjacent M-domain (Figure 3B). Accordingly, repressed variants do not interact with Hsp70 (Oguchi et al., 2012) and stabilization of the repressed state by the ClpB/Hsp104-specific inhibitor GdmHCl prevents Hsp70-dependent targeting of ClpB/Hsp104 to protein aggregates (Kummer et al., 2013).

Hsp70 binding will however stabilize a detached M-domain, leading to ClpB/Hsp104 activation. Evidence for such second function of Hsp70 next to ClpB/Hsp104 recruitment to protein aggregates has been provided (Seyffer et al., 2012; Lee et al., 2013; Rosenzweig et al., 2013). Mixing experiments of ClpB wild type and mutant subunits deficient in Hsp70 interaction indicate that a single Hsp70 encounter is not sufficient for ClpB activation. An initial binding of Hsp70 is, however, predicted to facilitate ClpB/Hsp104 interaction with a second Hsp70 chaperone, as the horizontal, repressed conformations of adjacent M-domains will be destabilized (Figure 3B).

Such mechanism would allow for a wavelike progression of ClpB/Hsp104 activation by subsequent Hsp70 binding events (Figure 3B). Close proximity of a second Hsp70 molecule is expected to exist at the surface of a protein aggregate but not for other, non-aggregated (e.g., nascent) polypeptide substrates, providing a molecular mechanism to restrict ClpB activity to protein aggregates. The model also predicts that a substrate dimer with two Hsp70 molecules bound could act as the smallest unit needed for ClpB activation, which indeed is the case for DnaK/ClpB-mediated monomerization of the dimeric replication initiation proteins RepA and TrfA (Konieczny and Liberek, 2002; Doyle et al., 2007).

ClpB/Hsp104 variants that permanently exist in the activated state exhibit an increased unfolding power and unfold stable domains during the disaggregation process, in contrast to ClpB/Hsp104 wild type (Oguchi et al., 2012). These hyperactive variants are superior to their ClpB/Hsp104 wild type counterparts in protein disaggregation (Oguchi et al., 2012; Lipinska et al., 2013) and activated Hsp104 variants can protect yeast cells from toxicity of neurodegenerative disease proteins including α-synuclein by reverting their aggregation into amyloidogenic deposits (Jackrel et al., 2014). This poses the question why such ClpB/Hsp104 variants were not selected for during evolution. Expression of activated ClpB/Hsp104 variants at higher levels or at increased temperatures is highly toxic, presumably because substrate threading occurs in an uncontrolled fashion, rationalizing the need for tight ClpB/Hsp104 activity control (Schirmer et al., 2004; Oguchi et al., 2012; Lipinska et al., 2013).

How does M-domain association and detachment control and change ClpB/Hsp104 ATPase activity? How the M-domain docking state signals to the ATPase center and which step in the ATPase cycle is modulated is currently unknown. Mixing experiments of ClpB/Hsp104 wild type and ATPase deficient subunits suggest that M-domain dissociation increases ATPase subunit cooperation and thus ATPase activity (Seyffer et al., 2012; Lee et al., 2013). More specifically, the ATPase domains of the activated state are primed for high ATP turnover, but require substrate binding as additional, second signal (Oguchi et al., 2012). This mechanism restricts high ClpB/Hsp104 ATPase and threading activity to the surface of protein aggregates.

## ClpB/Hsp104 N-domains increase disaggregation activities

The role of ClpB/Hsp104 N-domains in protein disaggregation has been elusive for a long time. N-domains are connected to the ClpB/Hsp104 ring via flexible linkers and appear highly mobile (Lee et al., 2003). Deleting N-domains does not inhibit ClpB disaggregation activity, however, single amino acid alterations of conserved N-domain residues abrogate disaggregation for unknown reasons (Beinker et al., 2002; Liu et al., 2002; Mogk et al., 2003). New findings implicate a supportive role of N-domains in protein disaggregation by increasing unfolding power. Fixing N-domain positioning by disulfide crosslinking reduces ClpB disaggregation activity, indicating that N-domain movements support substrate processing (Mizuno et al., 2012). Combining an N-domain deletion with pore loop mutations in AAA-1 abrogates disaggregation activity, whereas respective single alterations do not (Doyle et al., 2012). These findings suggest a role of N-domains in force generation and substrate engagement. Direct evidence for such function was recently provided by showing that ΔN-Hsp104 can partially process Sup35 NM fibers but cannot melt the central cross-β-structure, in contrast to Hsp104-wt (Sweeny et al., 2015). Accordingly, the ability of activated Hsp104 M-domain variants to protect yeast cells from toxic aggregation-prone proteins was lost upon N-domain deletion (Sweeny et al., 2015). N-domains likely directly contact substrates and their movements might cause shearing forces that add to the mechanical work done by ClpB/Hsp104.

## ClpB vs. Hsp104: fundamental differences in mechanism?

Despite significant progress in understanding Hsp70-Hsp100 cooperation the field has not reached a commonly accepted model of ClpB/Hsp104 mechanism and Hsp70 cooperation. It is controversially discussed whether bacterial ClpB and yeast Hsp104 differ in fundamental mechanistic aspects, including Hsp70-dependence and ATPase regulation. Hsp104 has been suggested to operate independently from Hsp70 and to gain unique activities in the propagation of protein-based heritable elements (prions) (Shorter and Lindquist, 2004; Desantis et al., 2012; Jackrel et al., 2014). Yeast prions result from the conversion of soluble proteins into ordered amyloid-like aggregates. Prion propagation involves several steps, starting from fibril growth via incorporation of soluble protein monomers, followed by the generation of seed templates (propagons) via fibril fragmentation and subsequent distribution to daughter cells during cell division (Shorter and Lindquist, 2005). Hsp104 appears to act primarily during fibril fragmentation *in vivo*, ensuring prion inheritance by the generation of propagons (Satpute-Krishnan et al., 2007). Hsp104 has been suggested to function differently in the solubilization of protein aggregates and the fragmentation of prion fibrils. Whereas, protein disaggregation requires Hsp70 cooperation, prion fiber severing does not (Shorter and Lindquist, 2004, 2006). Furthermore, Hsp104 is suggested to adapt its mode of ATP hydrolysis to the identity of bound substrate: ATP turnover proceeds in a probabilistic mode upon binding to stress-induced amorphous protein aggregates but switches to a coordinated mode upon interaction with amyloid fibers (Desantis et al., 2012). This operational plasticity was suggested to equip Hsp104 with a unique prion fibril severing activity, whereas ClpB function is restricted to protein aggregates.

The reported differences between ClpB and Hsp104 are unexpected as they share 45% sequence identity and the degree of sequence conservation is even higher for the ATPase domains. Accordingly, no major differences in ClpB/Hsp104 oligomer organization and positions of N- and M-domains were found in a comparative cryo EM analysis, providing no structural rationale for a change in mechanism (Carroni et al., 2014). The existence of a unique prion severing activity of Hsp104 was also challenged, by showing that ClpB can replace Hsp104 function in prion propagation in yeast cells (Reidy et al., 2012). Furthermore, the Sup35 NM prion can form infectious particles in *E. coli* cells and stable inheritance requires ClpB disaggregation function (Yuan et al., 2014). A unique Hsp104 activity in prion propagation is thus not apparent.

The reported Hsp70-independent activity of Hsp104 in prion severing is also inconsistent with *in vivo* findings from various laboratories. First, the propagation of several prions depends on Sis1, an Hsp40 co-chaperone of yeast Hsp70 (Higurashi et al., 2008; Hines et al., 2011; Reidy et al., 2014). Second, targeting of Hsp104 to prion aggregates in yeast cells requires Hsp70 activity (Winkler et al., 2012) and, accordingly, threading of prion molecules through the Hsp104 translocation channel requires upstream Sis1 activity (Tessarz et al., 2008; Tipton et al., 2008). Finally, Hsp104 was also reported to require additional cellular activities for prion fiber fragmentation *in vitro*, as it cannot act on its own (Inoue et al., 2004). While the identities of the required cellular factors were not determined in the latter study, it is tempting to speculate that these include Hsp70 and cooperating co-chaperones. The bulk evidence therefore suggests that ClpB and Hsp104 do not differ in mechanistic principles and that protein disaggregation and prion fiber fragmentation both rely on Hsp70-Hsp100 cooperation.

## Concluding remarks

Substantial progress has been made in the understanding of Hsp70 and Hsp100 cooperation during protein disaggregation. The model of M-domain mediated activity control and its modulation by Hsp70 is widely accepted. Competing models, however, still exist for Hsp70-Hsp100 interplay in prion fiber severing. Also, key aspects of Hsp70/Hsp100 cooperation and the regulation of Hsp100 ATPase cycle remain to be addressed. How can ClpB/Hsp104 outcompete nucleotide exchange factors for Hsp70 binding given the mediocre affinity between the Hsp70 and Hsp100 M-domains? Does the interaction with multiple Hsp70 increase affinity of ClpB/Hsp104 oligomers for the partner protein, providing a competitive advantage toward GrpE for Hsp70 binding? Does such mechanism enhance the selectivity of ClpB/Hsp104 toward protein aggregates? The allosteric regulation of the ATPase cycle of ClpB/Hsp104 is also far from being understood, due to its inherent high complexity. Furthermore, the mechanistic analysis so far focused on the regulation of the basal ClpB/Hsp104 ATPase activity, which is however changed upon cooperation with Hsp70 and thus during protein disaggregation. How ClpB/Hsp104 activation changes allosteric control is an important question that needs to be addressed by future work.

### Conflict of interest statement

The authors declare that the research was conducted in the absence of any commercial or financial relationships that could be construed as a potential conflict of interest.
